# Illegal Substance Use among Italian High School Students: Trends over 11 Years (1999–2009)

**DOI:** 10.1371/journal.pone.0020482

**Published:** 2011-06-10

**Authors:** Sabrina Molinaro, Valeria Siciliano, Olivia Curzio, Francesca Denoth, Stefano Salvadori, Fabio Mariani

**Affiliations:** Institute of Clinical Physiology, National Research Council, Pisa, Italy; Pennsylvania State University, United States of America

## Abstract

**Purpose:**

To monitor changes in habits in drug use among Italian high school students.

**Methods:**

Cross-sectional European School Survey Project on Alcohol and Other Drugs (ESPAD) carried out in Italy annually for 11 years (1999–2009) with representative samples of youth attending high school. The sample size considered ranges from 15,752 to 41,365 students and response rate ranged from 85.5% to 98.6%. Data were analyzed to obtain measures of life-time prevalence (LT), use in the last year (LY), use in the last 30 days (LM), frequent use. Comparisons utilized difference in proportion tests. Tests for linear trends in proportion were performed using the Royston p trend test.

**Results:**

When the time-averaged value was considered, cannabis (30% LT) was the most, and heroin the least (2%) frequently used, with cocaine (5%), hallucinogens (2%) and stimulants (2%) in between. A clear gender gap is evident for all drugs, more obvious for hallucinogens (average M/F LY prevalence ratio 2, range 1.7–2.4, p<0.05), less for cannabis (average M/F LY prevalence ratio 1.3, range 1.2–1.5, p<0.05). Data shows a change in trend between 2005 and 2008; in 2006 the trend for cannabis use and availability dropped and the price rose, while from 2005 cocaine and stimulant use prevalence showed a substantial increase and the price went down. After 2008 use of all substances seems to have decreased.

**Conclusions:**

Drug use is widespread among students in Italy, with cannabis being the most and heroin the least prevalent. Girls are less vulnerable than boys to illegal drug use. In recent years, a decrease in heroin use is overbalanced by a marked rise in hallucinogen and stimulant use.

## Introduction

In most industrialized countries the use of illegal psychoactive substances is a serious public health challenge, and usually begins during adolescence [1]. Thus, in all countries it is a public health imperative to assess the population rates of illicit drug use among adolescents. In addition, monitoring trends over time may reflect the net effects of activities and programs carried out to prevent adolescent substance use.

The European School Survey Project on Alcohol and Other Drugs (ESPAD) collects comparable data on substance use among European students in order to monitor trends within as well as between countries [2]. One of the most important and useful results from the ongoing series of ESPAD surveys is the estimation of changes taking place in the school population— changes in use of various drugs, in attitudes and beliefs that may help to explain changes in use, and within various demographic subgroups in the study population. The ESPAD study is also useful for assessing which new drugs or substances may be gaining favor, and in which subgroups or areas. This information has important implications for public policy—for assessing needs, setting agendas, and formulating and evaluating policies. More generally, it has implications for the health of the nation [3].

In our Institute, we carried out the Italian branch of the cross-sectional ESPAD study every year for 11 consecutive years (1999–2009) in representative samples of Italian youth attending school, yielding a continuous record of trends in drug use. Data collection was performed by standardized methodology using anonymous self-administered questionnaires completed in the classroom. Based on 11 consecutive years of a national school survey in Italy, this study aimed to examine the trend of cumulative and onset use of illegal drugs among school-age adolescents and the relationship between various measures of prevalence of illicit drug use and the perception of accessibility, prices of the illegal substances and changes in the drug laws and policies.

## Methods

### Procedure and participants

Data reported in this study are part of an ongoing longitudinal survey study by the Institute of Clinical Physiology of the Italian National Research Council. For this study, we used the data regarding eleven consecutive years, from ESPAD-Italia®1999 to ESPAD-Italia®2009 and prevalence of drug use was measured by identical instruments and methodology every year. The survey takes place every year in March–April; the survey assessments were self-administered using paper and pencil, requiring a duration of 40 minutes to complete. From 1999 a representative Italian student sample, aged 15–19 years, have been questioned about psychoactive substance use as well as leisure activities, relationships at school, attitude concerning drug use (approval or perceived risk), satisfaction with relationships with parents or friends, social and cultural status. General information about the sample, data collection and questionnaire is described in detail in Hibell et al. [2].

Repeating these cross-sectional studies over time allows an assessment of change across years in those same segments of the student population. Sample characteristics are reported in Table 1.

**Table 1:** Samples characteristics.

	1999	2000	2001	2002	2003	2004	2005	2006	2007	2008	2009
N	20185	22418	22257	15752	25299	32372	41365	38748	40407	38681	32461
Age (mean)	17.2±1.6	17.1±1.5	17.1±1.4	17.2±1.6	17.1±1.6	17.1±1.6	17.1±1.6	17.1±1.6	17.1±1.6	17.2±1.6	17.1±1.6
Gender (male)	41,8%	47,3%	45,0%	45,5%	45,5%	48,1%	48,1%	48,9%	49,7%	49,0%	49,2%
Response rate*	94,3%	92,2%	87,1%	98,6%	94,9%	96,1%	94,1%	88,9%	92,4%	85,8%	89,2%

*Response rate of schools participating in the survey.

### Measures

Drug use can be measured in terms of prevalence (the proportion of a defined population who have used a drug once or more in a particular time interval) or in terms of frequency (how many times they used the drug within a defined time interval). In this paragraph, both these important aspects of drug use are addressed in relation to each of the three time intervals considered in the ESPAD study — lifetime (LT), past 12 months (LY), past 30 days (LM), current frequent use (F) — utilizing data from the most recently completed cross-sectional surveys from high-school students, conducted in the spring. We also examine how prevalence of use varies across gender groups.

To analyze drug use among adolescents, information about lifetime, last year, and last month use variables were recorded by the answer to the questions “On how many occasions (if any) have you used …?” in the lifetime, last year and last month, with response categories: “never, once or twice, 3–5 times, 6–9 times, 10–19 times, 20–39 times and 40 times or more”.

In agreement with the item analyzed in Monitoring The Future Research [4], respondents were considered current frequent users if they indicated that they had used the drug on 20 or more occasions in the previous 30 days.

Data regarding lifetime use were collected from 1999 only for cannabis (marijuana or hashish), cocaine (also powder) and heroin (smoked and not), and from 2003 for hallucinogens (LSD and mushroom) and stimulants (GHB, ecstasy and amphetamines).

Data regarding use in the last year were collected from 1999 only for cannabis, from 2000 for cocaine and heroin, and from 2003 for hallucinogens and stimulants.

Data regarding use in the last month and frequent use were collected from 1999 only for cannabis, from 2002 for cocaine and heroin, and from 2003 for hallucinogens and stimulants.

One set of questions asks respondents how difficult they think it would be to obtain each of a number of different drugs if they wanted it. Perceived availability was dichotomized as very easy and fairly easy (coded as 1) vs other responses (fairly difficult; very difficult; impossible; don't know) [5].

Data on street costs of drugs were officially supplied by the Italian Interior Ministry [6].

### Statistical analysis

Prevalence with 95% confidence interval, for lifetime, last year, last month and frequent drug use were computed using Stata, version 10 for Windows (Stata Corp, 2001). Chi-square analyses were used to test these prevalences for gender differences. Statistical significance was set at p<0.05 (two-tailed). Tests for linear trend in proportion were performed using the Royston p trend test in the Stata module for trend analysis. Prevalence last year and last month use (except for frequent cannabis use) were also compared to determine whether the changes in prevalence of substance were related for change in perceived availability or in price. Short-term trends of each line segment were denoted by the percent change from first data available (i.e. for LY cannabis use the referent year is 1999, instead for cannabis street price 2001).

## Results

**Figure 1:**
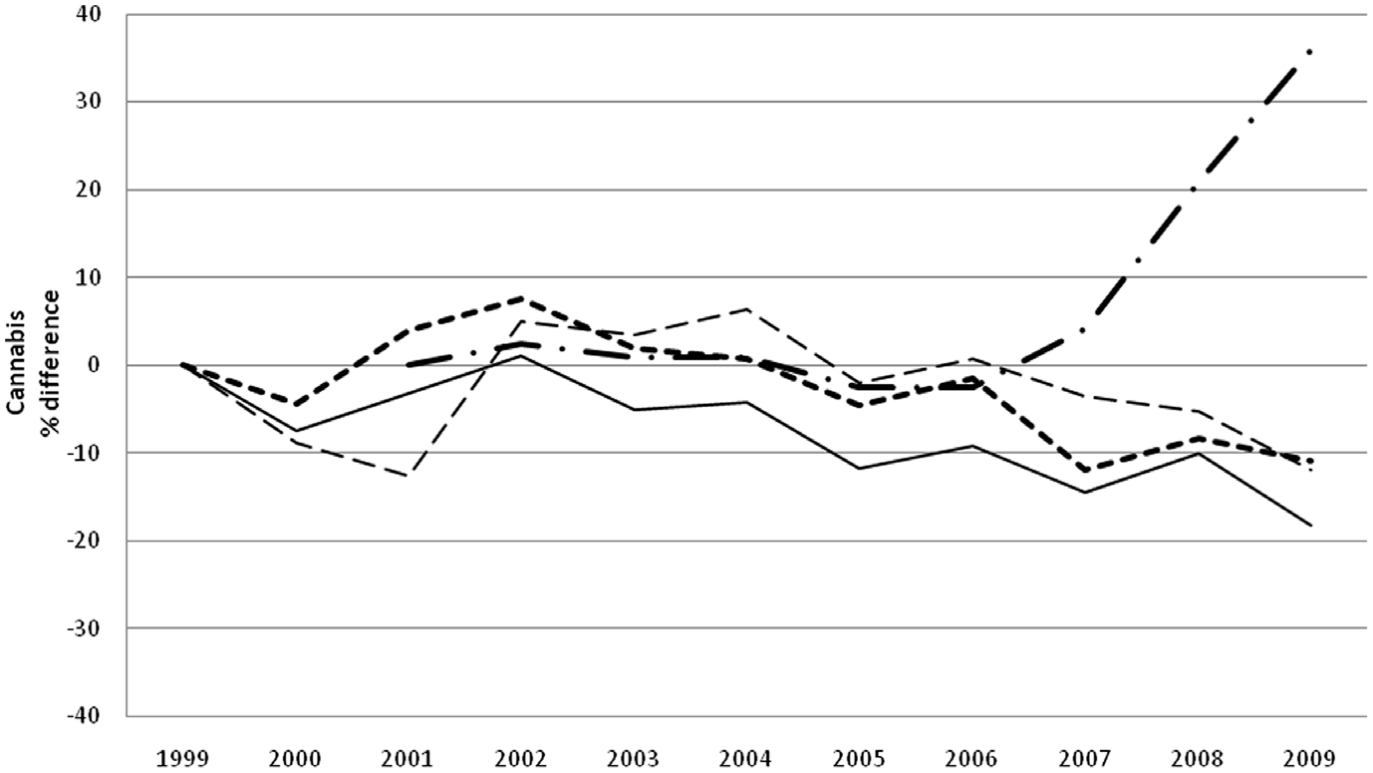
Cannabis. Percentage differences in LY and frequent cannabis use prevalence, in perceived cannabis availability prevalence and percentage differences in street price of cannabis.

**Figure 2:**
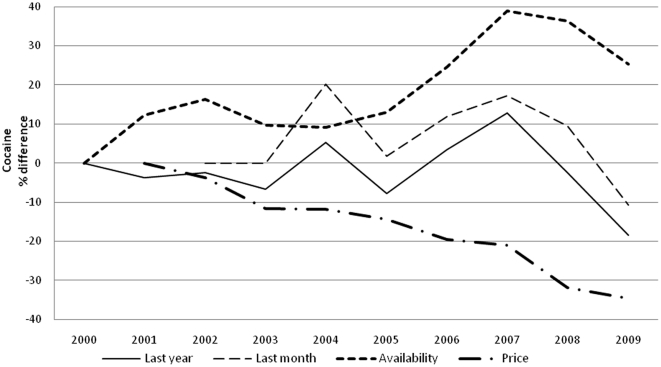
Cocaine. Percentage differences in LY and LM cocaine use prevalence, in perceived cocaine availability prevalence and percentage differences in street price of cocaine.

**Figure 3:**
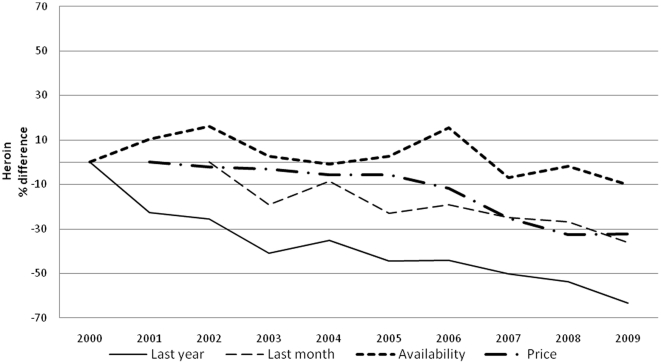
Heroin. Percentage differences in LY and LM heroin use prevalence, in perceived heroin availability prevalence and percentage differences in street price of heroin.

**Figure 4:**
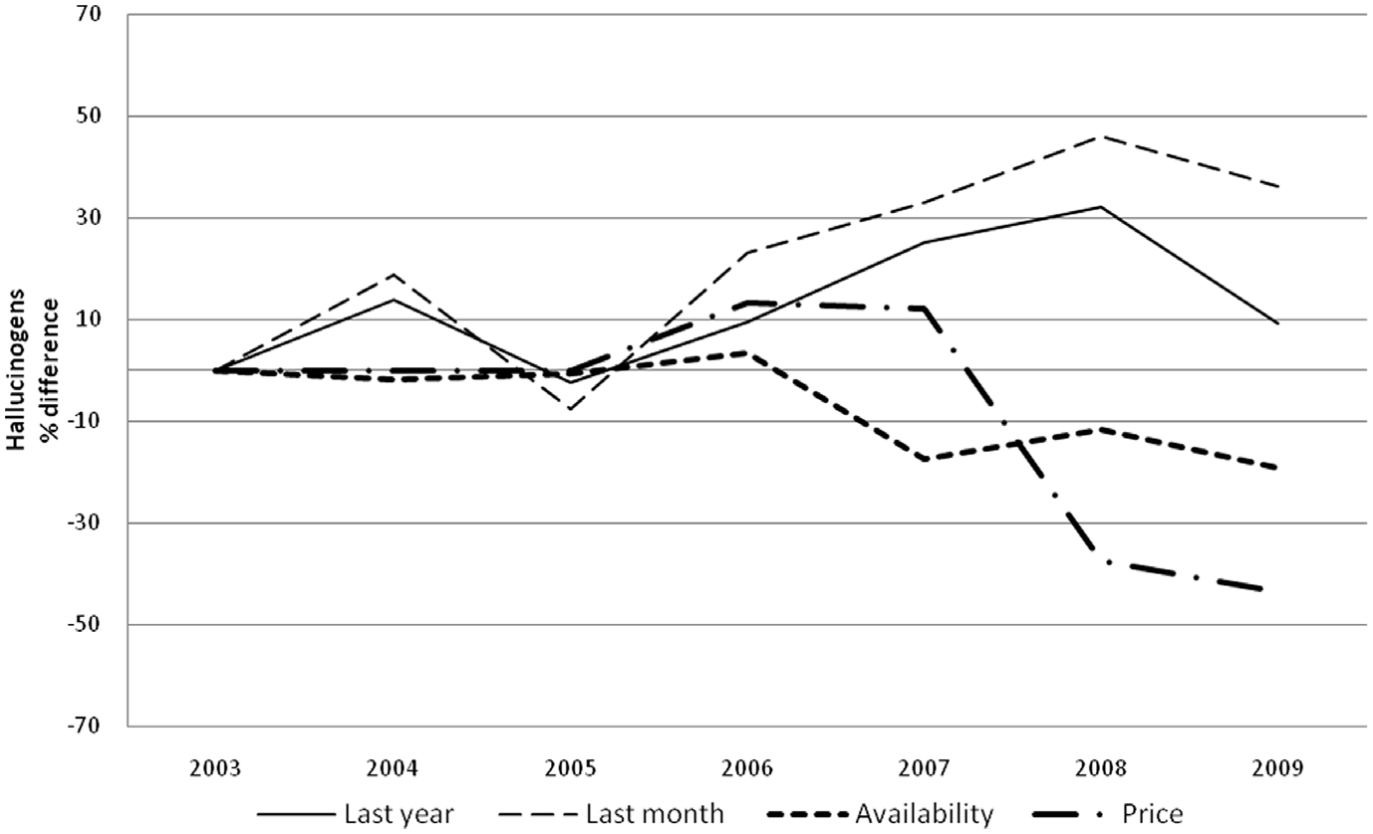
Hallucinogens. Percentage differences in LY and LM hallucinogen use prevalence, in perceived hallucinogen availability prevalence and percentage differences in street price of hallucinogens.

**Figure 5:**
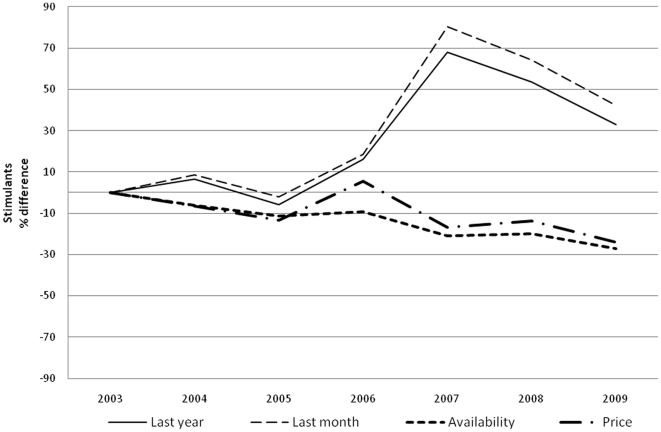
Stimulants. Percentage differences in LY and LM stimulant use prevalence, in perceived stimulant availability prevalence and percentage differences in street price of stimulants.

Prevalence of drug use is reported in Tables 2 and 3 for boys and girls respectively. For each year and gender, data are provided separately for each of the five major classes of illicit drugs. When the time-averaged value is considered, cannabis (over 30% of lifetime use) is the most, and heroin the least (LT use less than 3%) used, with cocaine (5%), hallucinogens and stimulants (4%) in between. A clear gender gap is evident for all drugs, more obvious for hallucinogens (average M/F LY prevalence ratio 2, range 1.7–2.4, p<0.05), stimulant (average M/F LY prevalence ratio 1.8, range 1.5–2.2, p<0.05) and cocaine (average M/F LY prevalence ratio 1.7, range 1.3–2.3, p<0.05) less for heroin (average M/F LY prevalence ratio 1.4, range 1.1–1.8, p<0.05) and cannabis (average M/F LY prevalence ratio 1.3 range 1.2–1.5, p<0.05). Over the years, LY cannabis use steady dropped (p<0.001) (Fig. 1), LY use of cocaine (Fig. 2) remained more or less stable, and LY heroin use(Fig. 3) decreased as well (p<0.001); whereas LY hallucinogen use (Fig. 4) and, more markedly, LY stimulant use (Fig. 5) increased (p<0.001). Regarding availability, cannabis has been the most consistently available illicit drug, but even it showed a small decrease over the years (Fig. 1), the same trend shown by hallucinogens and stimulants. Cocaine availability shows an increasing trend from 2006; 1 student in 5 reported easy accessibility of the drug. In the case of heroin availability, the situation is more stable.

**Table 2:** Prevalence of drug use (lifetime, last year, last month and frequent use) in Italian male students aged 15–19 years (%).

		1999	2000	2001	2002	2003	2004	2005	2006	2007	2008	2009	p trend
Cannabis	Lifetime	37.2 (36.1;38.2)	34.4 (33.5;35.3)	34.8 (33.9;35.7)	39.0 (37.9;40.2)	37.0 (36.1;37.9)	35.9 (35.1;36.7)	33.7 (33.0;34.4)	35.6 (34.9;36.3)	32.9 (32.3;33.6)	35.1 (34.5;35.8)	33.8 (33.0;34.5)	***
	Last year	30.2 (29.2;31.1)	29.2 (28.3;30.1)	28.6 (27.7;29.5)	32.0 (30.9;33.1)	30.2 (29.3;31.0)	29.4 (28.7;30.1)	26.7 (26.0;27.3)	27.9 (27.3;28.6)	26.7 (26.1;27.4)	27.9 (27.2;28.5)	26.7 (26.0;27.4)	***
	Last month	20.8 (19.9;21.7)	19.6 (18.8;20.3)	18.8 (18.1;19.6)	21.5 (20.6;22.5)	20.6 (19.9;21.4)	19.9 (19.2;20.5)	17.6 (17.0;18.1)	18.3 (17.7;18.9)	18.0 (17.5;18.6)	18.4 (17.9;19.0)	17.6 (17.0;18.2)	***
	Frequent	4.6 (4.1;5.0)	3.7 (3.3;4.1)	3.6 (3.2;4.0)	4.7 (4.2;5.2)	4.6 (4.2;5.0)	4.5 (4.2;4.9)	4.0 (3.7;4.3)	4.2 (3.9;4.5)	4.1 (3.8;4.3)	4.1 (3.8;4.4)	3.9 (3.6;4.2)	n.s.
Cocaine	Lifetime	7.3 (6.7;7.8)	6.1 (5.6;6.6)	6.3 (5.8;6.7)	7.5 (6.9;8.2)	7.1 (6.6;7.6)	6.6 (6.2;7.0)	6.6 (6.2;6.9)	6.8 (6.4;7.2)	7.6 (7.2;8.0)	7.2 (6.8;7.5)	6.2 (5.8;6.6)	n.s.
	Last year		5.0 (4.5;5.4)	4.1 (3.7;4.5)	5.0 (4.5;5.5)	4.8 (4.4;5.2)	4.7 (4.4;5.1)	4.4 (4.1;4.7)	4.8 (4.5;5.1)	5.3 (5.0;5.6)	4.7 (4.4;5.1)	4.3 (4.0;4.6)	n.s.
	Last month				2.3 (2.0;2.7)	2.4 (2.1;2.7)	2.3 (2.1;2.6)	2.3 (2.1;2.5)	2.4 (2.2,2.6)	2.6 (2.4;2.9)	2.4 (2.2;2.7)	2.2 (2.0;2.4)	n.s.
	Frequent				0.2 (0.1;0.4)	0.2 (0.1;0.3)	0.2 (0.1;0.3)	0.3 (0.2;0.3)	0.2 (0.2;0.3)	0.4 (0.3;0.4)	0.5 (0.4;0.6)	0.7 (0.6;0.8)	***
Heroin	Lifetime	4.1 (3.7;4.6)	4.6 (4.2;5.0)	3.6 (3.2;3.9)	3.9 (3.5;4.4)	3.0 (2.7;3.3)	2.8 (2.6;3.1)	2.7 (2.4;2.9)	2.9 (2.7;3.1)	2.4 (2.1;2.6)	2.4 (2.2;2.6)	2.1 (1.8;2.3)	***
	Last year		3.2 (2.9;3.6)	2.5 (2.2;2.8)	2.5 (2.1;2.9)	1.9 (1.6;2.1)	2.0 (1.7;2.2)	1.7 (1.5;1.8)	1.8 (1.6;2.0)	1.6 (1.4;1.8)	1.6 (1.4;1.8)	1.3 (1.1;1.5)	***
	Last month				1.4 (1.1;1.7)	1.1 (0.9;1.3)	1.1 (0.9;1.3)	1.0 (0.8;1.1)	1.0 (0.8;1.1)	1.0 (0.8;1.1)	1.0 (0.9;1.1)	1.0 (0.9;1.2)	*
	Frequent				0.2 (0.1;0.3)	0.1 (0.1;0.2)	0.1 (0.1;0.2)	0.2 (0.1;0.2)	0.2 (0.1;0.3)	0.3 (0.3;0.4)	0.3 (0.3;0.4)	0.5 (0.4;0.7)	***
Hallucinogens	Lifetime					5.0 (4.6;5.4)	5.5 (5.2;5.9)	5.1 (4.8;5.4)	5.3 (5.0;5.6)	5.6 (5.3;5.9)	5.9 (5.6;6.3)	5.3 (5.0;5.7)	*
	Last year					3.1 (2.7;3.4)	3.1 (2.9;3.4)	2.8 (2.6;3.1)	3.3 (3.0;3.5)	3.6 (3.3;3.8)	3.8 (3.5;4.1)	3.4 (3.1;3.7)	***
	Last month					1.3 (1.1;1.5)	1.4 (1.2;1.5)	1.2 (1.1;1.4)	1.5 (1.3;1.7)	1.6 (1.4;1.8)	1.8 (1.6;2.0)	1.8 (1.6;2.0)	***
	Frequent					0.2 (0.1;0.3)	0.1 (0.0;0.1)	0.2 (0.1;0.2)	0.3 (0.2;0.3)	0.4 (0.3;0.5)	0.5 (0.4;0.6)	0.7 (0.5;0.8)	***
Stimulants	Lifetime					4.3 (3.9;4.6)	3.8 (3.5;4.1)	3.8 (3.6;4.1)	4.3 (4.0;4.6)	5.7 (5.4;6.0)	5.7 (5.3;6.0)	5.2 (4.9;5.6)	***
	Last year					2.6 (2.3;2.9)	2.4 (2.1;2.6)	2.2 (2.0;2.4)	2.8 (2.5;3.0)	4.0 (3.7;4.2)	3.7 (3.4;3.9)	3.4 (3.1;3.7)	***
	Last month					1.3 (1.1;1.5)	1.2 (1.0;1.3)	1.2 (1.0;1.3)	1.4 (1.2;1.5)	2.2 (2.0;2.4)	2.0 (1.8;2.2)	1.9 (1.7;2.1)	***
	Frequent					0.2 (0.1;0.2)	0.1 (0.0;0.1)	0.2 (0.1;0.2)	0.2 (0.1;0.3)	0.5 (0.4;0.6)	0.5 (0.4;0.6)	0.8 (0.6;0.9)	***

*** p<0.001 **p<0.01 * p<0.05.

**Table 3:** Prevalence of drug use (lifetime, last year, last month and frequent use) in Italian female students aged 15–19 years (%).

		1999	2000	2001	2002	2003	2004	2005	2006	2007	2008	2009	p trend
Cannabis	Lifetime	30.3 (29.5;31.1)	27.6 (26.7;28.4)	30.7 (29.8;31.5)	30.0 (29.0;30.9)	28.2 (27.5;29.0)	29.3 (28.6;30.0)	28.0 (27.3;28.6)	28.7 (28.0;29.3)	25.6 (25.0;26.3)	28.0 (27.3;28.6)	24.7 (24.1;25.4)	***
	Last year	24.7 (23.9;25.5)	21.0 (20.3;21.8)	24.0 (23.2;24.7)	23.2 (22.3;24.2)	21.8 (21.1;22.5)	22.5 (21.8;23.1)	21.1 (20.6;21.7)	21.2 (20.6;21.8)	19.4 (18.8;19.9)	20.8 (20.2;21.3)	17.5 (16.9;18.1)	***
	Last month	14.4 (13.7–15.0)	13.3 (12.7;13.9)	14.4 (13.7;15.0)	13.8 (13.0;14.5)	12.9 (12.2;13.5)	13.4 (12.9;13.9)	12.7 (12.3;13.2)	12.4 (11.9;12.9)	11.2 (10.8;11.7)	12.2 (11.7;12.6)	9.7 (9.2;10.1)	***
	Frequent	1.6 (1.4;1.8)	1.6 (1.3;1.8)	1.6 (1.3;1.8)	1.6 (1.3;1.8)	1.6 (1.4;1.8)	1.6 (1.4;1.8)	1.7 (1.5;1.9)	1.6 (1.4;1.8)	1.5 (1.3;1.6)	1.3 (1.2;1.5)	1.1 (1.0;1.3)	***
Cocaine	Lifetime	*3.5 (3.2;3.9)*	*3.6 (3.2;3.9)*	*4.7 (4.3;5.0)*	*4.0 (3.5;4.4)*	*3.7 (3.4;4.0)*	*4.3 (4.0;4.6)*	*4.1 (3.8;4.4)*	*4.4 (4.1;4.6)*	*5.0 (4.7;5.3)*	*4.5 (4.2;4.8)*	*3.4 (3.1;3.7)*	**
	Last year		*2.7 (2.4;3.0)*	*3.2 (2.9;3.5)*	*2.5 (2.2;2.9)*	*2.5 (2.2;2.7)*	*3.2 (3.0;3.5)*	*2.6 (2.4;2.8)*	*3.0 (2.7;3.2)*	*3.2 (3.0;3.4)*	*2.6 (2.2;2.8)*	*1.9 (1.7;2.1)*	**
	Last month				*1.2 (0.9;1.4)*	*1.1 (0.9;1.3)*	*1.7 (1.5;1.9)*	*1.2 (1.1;1.4)*	*1.4 (1.2;1.6)*	*1.3 (1.2;1.5)*	*1.3 (1.1;1.5)*	*0.9 (0.7;1.0)*	**
	Frequent				*0.1 (0.0;0.2)*	*0.1 (0.0;0.1)*	*0.1 (0.1;0.2)*	*0.1 (0.0;0.1)*	*0.1 (0.1;0.2)*	*0.1 (0.1;0.2)*	*0.2 (0.1;0.2)*	*0.2 (0.1;0.3)*	***
Heroin	Lifetime	*2.9 (2.6;3.2)*	*3.2 (2.8;3.5)*	*2.9 (2.6;3.1)*	*2.6 (2.3;2.9)*	*2.4 (2.1;2.7)*	*2.4 (2.2;2.7)*	*2.3 (2.1;2.5)*	*2.2 (2.0;2.4)*	*2.1 (1.9;2.3)*	*1.8 (1.6;2.0)*	*1.4 (1.2;1.5)*	***
	Last year		*2.4 (2.1;2.7)*	*1.8 (1.6;2.1)*	*1.7 (1.5;2.0)*	*1.5 (1.3;1.7)*	*1.7 (1.5;1.9)*	*1.5 (1.3;1.6)*	*1.3 (1.2;1.5)*	*1.2 (1.0;1.3)*	*1.0 (0.9;1.1)*	*0.7 (0.6;0.9)*	***
	Last month				*0.8 (0.6;0.9)*	*0.7 (0.5;0.8)*	*0.8 (0.7;1.0)*	*0.7 (0.6;0.8)*	*0.7 (0.6;0.9)*	*0.6 (0.5;0.7)*	*0.6 (0.5;0.7)*	*0.4 (0.3;0.5)*	***
	Frequent				*0.1 (0.0;0.1)*	*0.1 (0.0;0.1)*	*0.1 (0.0;0.1)*	*0.1 (0.0;0.1)*	*0.1 (0.1;0.2)*	*0.2 (0.1;0.2)*	*0.1 (0.1;0.2)*	*0.2 (0.1;0.2)*	***
Hallucinogens	Lifetime					*2.6 (2.4;2.9)*	*3.1 (2.9;3.4)*	*2.8 (2.6;3.1)*	*2.8 (2.6;3.0)*	*3.3 (3.0;3.5)*	*3.5 (3.3;3.8)*	*2.6 (2.4;2.9)*	*n.s.*
	Last year					*1.5 (1.3;1.7)*	*1.9 (1.7;2.1)*	*1.5 (1.3;1.6)*	*1.6 (1.4;1.7)*	*1.9 (1.7;2.1)*	*2.0 (1.8;2.2)*	*1.4 (1.2;1.6)*	*n.s.*
	Last month					*0.6 (0.4;0.7)*	*0.8 (0.7;1.0)*	*0.5 (0.4;0.6)*	*0.8 (0.6;0.9)*	*0.8 (0.7;0.9)*	*0.9 (0.7;1.0)*	*0.7 (0.6;0.8)*	*
	Frequent					*0.1 (0.0;0.1)*	*0.0 (0.0;0.1)*	*0.1 (0.0;0.1)*	*0.1 (0.1;0.2)*	*0.1 (0.1;0.2)*	*0.2 (0.1;0.3)*	*0.2 (0.1;0.2)*	***
Stimulants	Lifetime					*2.3 (2.1;2.6)*	*2.8 (2.5;3.0)*	*2.3 (2.1;2.5)*	*2.5 (2.3;2.8)*	*3.8 (3.5;4.0)*	*3.8 (3.5;4.1)*	*3.1 (2.8;3.3)*	***
	Last year					*1.2 (1.0;1.4)*	*1.6 (1.4;1.8)*	*1.3 (1.1;1.4)*	*1.6 (1.4;1.8)*	*2.3 (2.1;2.5)*	*2.1 (1.9;2.3)*	*1.5 (1.3;1.7)*	***
	Last month					*0.6 (0.5;0.7)*	*0.9 (0.7;1.0)*	*0.6 (0.5;0.7)*	*0.8 (0.7;0.9)*	*1.1 (1.0;1.3)*	*1.0 (0.9;1.2)*	*0.8 (0.6;0.9)*	***
	Frequent					*0.1 (0.0;0.1)*	*0.1 (0.0;0.1)*	*0.1 (0.0;0.1)*	*0.1 (0.1;0.2)*	*0.2 (0.1;0.3)*	*0.2 (0.1;0.2)*	*0.2 (0.1;0.3)*	***

*** p<0.001 **p<0.01 * p<0.05.

## Discussion

Our study shows that illicit drug use is a widespread and probably expanding epidemic among Italian high school students, with cannabis still at least five times more prevalent than any other drug. Boys are more vulnerable than girls to drug use. Drug consumption also shows a dynamic evolution over time, possibly modulated by cultural, political and economic factors, such as changing laws and variability of market prices. In spite of conspicuous legislative and social communication efforts in the field by various governments in the last 10 years, the prevalence of drug use was remarkably stable for the most commonly used drugs such as cannabis and cocaine, with a decrease in heroin overbalanced by a marked rise in hallucinogen and stimulant use. Data shows a change in trend between 2005 and 2008; in 2006 the trend for cannabis use and availability dropped and its price rose (Fig. 1), while from 2005 cocaine and stimulant use prevalence showed a substantial increase and the price went down (Figs. 2 and 4). After 2008 the use of all substances seems to have decreased.

### Study relevance

The findings of this study have social, medical and possibly legislative implications. The long-term adverse health consequences of illicit drug use are well documented, but short-term outcomes among adolescents are also important and include association with injury, violence and suicide, teenage pregnancy, sexually transmitted diseases, and poor mental health [7]. There is increasing concern about drug use during adolescence, since brain development during this period is more vulnerable to drug-related deficits [8]. At the public health level, the large proportion of adolescents who misuse psychoactive substances calls for more effective intervention strategies as well as better perception by politicians and decision makers of the seriousness and complexity of this issue [1], [9], [10], [11], [12].. The legislative climate in Italy has recently changed, with more stringent control of illegal drug use. The current 2006 law (L 49/2006) modified the previous one of 1990 (DPR 309/1990). The new regulatory framework was characterized by stiffer penalties in relation to the production, trafficking, possession and use of drugs, and by the abolition of any distinction between different kind of illicit drugs. Two hypotheses have been advanced to explain this mismatch between increased awareness of the problem by policy-makers, conspicuous legislative and communication efforts and lack of commensurate results. First, many preventive programs, especially in the school setting, are run as one-shot interventions, without a long-term link to parents and the surrounding community. Successful projects in the field tend to emphasize life skills and the participation of young people and parents/communities [13]. Second, social communication and legislative measures can see their effects minimized and even nullified by the phenomenon of cognitive dissonance [14]. If not recognized and properly handled, the emotional state of dissonance – which occurs when there is inconsistency between two cognitions or between a cognition and a behavior - is a strong barrier to changing behaviors in several health-related situations, including substance abuse and prevention of addiction, recently shown for instance in effective attempts to reduce adolescents' overuse of online gaming [15].

### Comparison with previous studies

Our data are in agreement with previous studies showing the large prevalence of adolescent drug use in Europe [11], [16]–[20] with greater vulnerability of boys compared to girls [17], [18], [21]. Others have previously shown the relative decline of heroin and the growth of hallucinogens and stimulants [19], [20]. Several studies provide a picture of adolescent drug use [17], [18], [22], [23] but no representative study has been conducted in Italy. In addition, compared to the available studies this has the largest sample and longest follow-up [24].

### Study limitations

In our study, each of the indicators used for drug use, drug availability, and cost has several limitations.

The survey approach with self-administered paper and pencil questionnaire is costly, time-consuming and requires cooperation from school officials. As in all similar surveys, percentages must be interpreted with caution as these are self-reported values. Survey measurements of such highly sensitive or stigmatized behaviors may generate inaccurate reporting and bias in survey estimates. A discussion of potential biases in self-reporting of substance use is provided elsewhere [11], [25], [26]. School-based surveys provide prevalence estimates of substance use, but do not capture street and homeless youths and other high-risk adolescents not found in the school environment [27]. School attendance is irregular or absent, and this subgroup is at a higher risk of illegal drug involvement [1], [22], [28], [29].

We use the term “perceived availability” when discussing availability because it is the person's perception that is being measured [23]. We recognize that availability is multidimensional, and respondents may consider a variety of factors in their answers, including knowing where to get access, difficulty getting to an access place, and possibly even monetary cost. However, we suspect that for most respondents, what we are measuring is perceived access, with little or no consideration of monetary cost.

While no systematic effort has been made to directly assess the validity of these measures (since such an assessment would involve actual attempts to obtain drugs), it must be said that the measures do have a fairly high level of face validity, particularly since it is the subjective reality of perceived availability being measured. It also seems reasonable to assume that to a considerable extent, perceived availability tracks actual availability. In addition, differences in reported availability across drugs, which generally correspond to reported prevalence of use, provide further evidence of validity.

To place the data within a wider economic context, in the results we also included rough data of drug prices, supplied by the Interior Ministry. Over the years, in Italy there has been a strong and substantial decrease in illegal drug prices at street level. Data supplied by the Interior Ministry show that between 2001 and 2009, the minimum prices for cocaine, heroin (both white and brown sugar), MDMA and LSD have decreased by over 30%. A 50% reduction was observed for LSD, from about 27 €/dose to just over 14 €/dose. Cocaine cost dropped from around € 90/g to about 59 €/g, white heroin from 78.5 €/g to about 53 €/g, MDMA from about 22 €/tablet to about 15 €/tablet. The price of marijuana and hashish remained stable during the observation period, just below 9 €/g for both [6]. Although large regional variations in true price can occur, there is little doubt that these economic indicators should be taken into account when considering trends in use.

### Conclusions

Drug use is widespread among high-school students in Italy, with cannabis being the most and heroin the least prevalent. Girls are less vulnerable than boys to illegal drug use. In recent years, a decrease in heroin use is overbalanced by a marked rise in hallucinogen and stimulant use. Despite the fairly large number of legislative and social communication initiatives for the prevention of substance abuse in our country, the situation has not yet improved to any significant degree.
